# Birth plan alterations among American women in response to COVID‐19

**DOI:** 10.1111/hex.13077

**Published:** 2020-05-24

**Authors:** Theresa E. Gildner, Zaneta M. Thayer

**Affiliations:** ^1^ Department of Anthropology Dartmouth College Hanover NH USA

To the editor:

The COVID‐19 pandemic has drastically strained the American healthcare system.^1^ Crowded hospitals, overworked staff and a lack of medical equipment have implications for those needing medical care unrelated to COVID‐19, including pregnant women. However, few studies have examined the impact of the pandemic on maternity care. What little work has been done has predominantly focused on treatment of pregnant women suffering from COVID‐19^2^, ^3^ and the risk of virus transmission from mother to baby.^3^, ^4^, ^5^, ^6^

It remains unclear how the pandemic has influenced maternal care choices, in particular how women have altered their birth plans. Data on common birth plan changes are needed to help providers better understand factors shaping care decisions, information that can be used to address patient concerns and tailor care recommendations. Here, we use an online convenience survey to assess how American women's birth plans (eg intended labour support and delivery location) have changed in response to the COVID‐19 pandemic.

The COVID‐19 And Reproductive Effects (CARE) study was posted on social media platforms (Facebook, Twitter) and distributed via email to contacts working in maternity care and public health. Pregnant women over the age of 18 and living in the United States were invited to participate in a survey assessing how the pandemic was impacting their medical care and birth plans. The data presented here were collected between 16 and 20 April 2020.

In addition to providing basic demographic data, gestational week and intended delivery facility, participants were asked whether ‘any aspect of your birth plan changed due to COVID‐19’ (yes/no). If they answered yes, they were then asked why and how their plans changed. These analyses were limited to participants who completed the entire survey (n = 1400). A subset of this sample also answered an open‐ended question describing the specific changes to their birth plans (n = 592); these qualitative responses were assessed for common patterns.^7^ This study received ethical approval from Dartmouth College (STUDY00032045).

The average participant age was 31.4 years old. The majority of participants were married/in domestic partnerships (94.5%), White (85.9%), had completed at least a bachelor's degree (77.3%), were employed full‐time (61.1%) and reported having not experienced any COVID‐19‐like symptoms (89.4%). Most participants planned on giving birth in a hospital (94.8% in hospital, 2.9% in free‐standing birth centres, 2.3% at home).

Overall, 45.2% of respondents reported changing some aspect of their birth plans because of COVID‐19, due in part to their own concerns (53.9% of respondents), their partner's concerns (21.9%), the concerns of friends or family members (13.8%) and comments from medical providers (60.8%). The prevalence of women changing their birth plans varied by trimester. Approximately 28.6% of women in their first trimester (36/126) reported changing their plans due to COVID‐19, compared to 37.0% of women in their second trimester (228/617 women) and 56.2% of women in their third trimester (369/657 women).

Commonly provided COVID‐related birth plan changes—from a subset of 592 respondents—are outlined in Table 1. These changes largely fell into three categories: (i) modifying an existing hospital birth plan (ie shortening the hospital stay, altered pain management strategies and accommodating new policies like wearing a mask while labouring); (ii) changing birth locations and/or providers (ie opting for an out of hospital birth, forced provider/location change because pandemic has limited availability, selecting hospital birth because of fear complications); and (iii) other COVID‐related concerns (ie having fewer support people at birth, visitors not permitted following birth, care disrupted because moved in response to shelter‐in‐place orders).

**Table 1:** Commonly reported reasons for birth plan changes (eg changes in birth location, labour and delivery preferences) provided by a subset of the sample (n = 592)

Remaining at same hospital with altered birth plan	Changing birth locations due to the pandemic	Other COVID‐related concerns
Planning to shorten my hospital stay and reduce risk of exposure by scheduling a C‐section, planning an induction, labouring at home as long as possible before transferring to hospital to deliver, and/or reducing post‐delivery recovery time (n = 62)	No longer plan for hospital birth because I worry about being separated from my newborn, having to labour alone, and/or I fear COVID‐19 exposure (n = 81)	Will have fewer people present to support me during delivery (and they will not be allowed to leave and return) because of delivery room restrictions and/or I fear that they have been exposed to the virus (n = 367)
Will have to accommodate new hospital protective policies during delivery (eg masks on labouring mothers and restricted movement during labour) (n = 46)	The pandemic has restricted provider availability and/or deliveries at local birth centres no longer allowed (n = 25)	Loved ones can no longer travel to visit after the birth and/or my partner may have to care for our other children and not be able to support me in labour (n = 186)
Altered pain management strategy (eg nitrous oxide no longer offered, worried to get epidural because of exposure risk, or can no longer have a water birth) (n = 42)	Now prefer hospital birth over home birth in case things go wrong: do not want to suffer complications because unknowingly have virus or have trouble getting admitted because hospital at capacity (n = 8)	Left COVID‐19 epicentre and/or temporarily moved in with family to shelter‐in‐place and now have to find a new provider (n = 8)

Note

The number of responses for each COVID‐related reason is noted, respondents sometimes listed more than one factor impacting their birth decisions.

Many participants planned to remain with their current provider, but were preparing for a very different birth experience with fewer support persons during labour (n = 367/592) and no visitors to meet their baby or care for their other children during delivery (n = 186/592). Some women worried they might have to labour alone if childcare could not be arranged and their partner had to watch their other children instead of attending the birth. Several women reported opting to no longer give birth in a hospital due to restrictions on who would be allowed in the delivery room, the possibility of forced separation from their newborn and fear of virus exposure (n = 81/592).

Pregnant American women face a variety of care‐related challenges related to COVID‐19, and our results demonstrate that this has led to altered birth plans. Yet, the reasons behind the changes appear to be individual‐dependent. While several participants reported that they now preferred an out of hospital birth because they feared COVID‐19 exposure, a handful of women reported choosing a hospital instead of a home birth because they feared suffering COVID‐related complications during delivery if they were unknowing carriers, or because they worried they would not be admitted to an overcrowded hospital should they need to transfer.

Notably, the women in this sample exhibited a much higher preference for out of hospital births than the national average before the COVID‐19 pandemic (5.4% vs. 1.6%, respectively).^8^ While this may be due in part to the demographic characteristics of the sample, this percentage is also comparatively high among women in the sample who had previously given birth. Specifically, of the 667 women in the sample who had previously given birth, 3.1% reported an out of hospital delivery for at least one of their previous births, but 5.1% of these same women now reported they planned for an out of hospital delivery. This, combined with the qualitative data reported here, suggests that part of this increase in out of hospital births is likely attributable to the COVID‐19 pandemic.

It is important to note that due to the use of convenience sampling these data are not representative of the whole US population. Additional work is needed using more diverse samples to identify additional issues influencing maternal care decisions. The diverse factors influencing maternal birth plan choices highlighted here may serve as reference providers can use to explore the specific concerns of each patient, ultimately leading to more productive provider‐patient conversations about the maternal care and delivery options available to best address any given situation.

## CONFLICT OF INTEREST

The authors declare that they have no competing interests.

## Data Availability

Data are available from the corresponding author upon reasonable request.
